# Sustainability stories in Swedish riding schools: an ecolinguistic analysis of policy documents and interviews

**DOI:** 10.3389/fspor.2026.1821456

**Published:** 2026-10-14

**Authors:** Petra Andersson, Anna Sätre, Gabriella Torell Palmquist, Aage Radmann, Susanna Hedenborg

**Affiliations:** 1 Department of Philosophy, Linguistics and Theory of Science, University of Gothenburg, Goteborg, Sweden; 2 Department of Sport Science, Malmo University, Malmo, Sweden; 3 Department of Educational Studies, Karlstad University, Karlstad, Sweden; 4 The Swedish Equestrian Centers of Excellence - Ridskolan Strömsholm & Flyinge AB, Strömsholm, Sweden; 5 Department of Teacher Education and Outdoor Studies, Norwegian School of Sport Sciences, Oslo, Norway

**Keywords:** ecolinguistics, equine sector, policy, practice, riding schools, sustainability

## Abstract

The equine sector provides employment opportunities and leisure activities for a substantial number of people in Sweden. Although these activities are closely connected to nature and dependent on ecosystem services, the sector faces significant ecological challenges. This article explores how representatives of the equine sector—particularly riding schools—conceptualize and articulate their work with environmental sustainability in policy documents and “talk about” everyday practice. The analysis is informed by the Sustainable Development Goals (SDGs) and grounded in an ecolinguistic framework. Our results show that national level policy documents are closely aligned with the established sustainability terminology, whereas local documents and interviewees rarely employ SDG related terminology. Despite this discursive distance, riding schools demonstrate strong engagement and considerable creativity in addressing local environmental challenges. However, this ambition is constrained by limited knowledge, resources, and authority to address more complex ecological issues. Within the pedagogical environments that riding schools constitute, systematic instruction on environmental sustainability holds a marginal position. We identified five “stories we live by”: “the responsible local care taker”, “we try, but we lack resources”, “learning through everyday practice”, “sustainability without sustainability terminology” and “small action matters”. We have interpreted this as emerging eco-benifical narratives that can carry pedagogical potential. We argue that with strengthened organizational support, clearer conceptual tools, and structured opportunities for capacity building, riding schools could play a more active role in shaping sustainability-oriented “stories to live by” and contribute more fully to the broader societal transition toward sustainable development.

## 1 Introduction

Compared to many other countries, Sweden has a notably high number of horses per capita. Horse riding is a popular leisure activity among both children and adults—particularly among girls and women. Equestrian sports, harness racing and flat racing attract substantial audiences, and racing is coupled with considerable betting activity. In addition, horses play an important role in various equine-assisted interventions, including educational, physiotherapeutic, and social work practices (1, 2).

Most horses in Sweden are kept near major cities or in densely populated areas, as owners prefer to keep their horses close to where they live. Consequently, equestrian activities place pressure on access to peri urban land (3). While horse related activities depend on ecosystem services and, under favorable conditions, can contribute positively to biodiversity and ecosystem functioning (4), they sometimes pose sustainability challenges in these areas. Horses require extensive pastureland as well as the production of straw and grain, and they generate large volumes of manure that, although potentially valuable as fertilizer, frequently become a waste management problem. The proximity to urban areas limits access to surrounding farmland capable of absorbing the quantities produced (5). Moreover, horse manure may contribute to nutrient leakage (6). In 2020, a Swedish government report on eutrophication (7) sparked considerable debate within the equine sector, particularly concerning nutrient leakage from paddocks and insufficient manure management (8).

The urgency of global climate and environmental challenges underscores the need for rapid action, yet the equine sector is often portrayed as insufficiently engaged in environmental issues or “green” practices. It has even been labelled a “climate villain”, with estimates suggesting that the sector emits more carbon dioxide than domestic air travel (9). Although the equine sector appears closely connected to nature and is dependent on ecosystem services, environmental awareness has traditionally been low—or at least perceived as such (10, 11). This pattern is not unique to the equine sector. On the contrary, reviews indicate that further research on sustainability within the broader leisure sector is needed (12). Research on sports and outdoor activities suggests that one obstacle to enhancing ecological sustainability among athletes is that their motivations for participation are primarily grounded in performance and training logics rather than in an interest in environmental sustainability. Participation in outdoor activities tends to be more strongly associated with an interest in preserving nature. However, these activities frequently require transportation—often by car—to locations where participation is possible (13, 14). Additionally, studies highlight that substantial variation exists between different activities within both the sports sector and the outdoor recreation sector (13). To date, research on these issues within the equine sector remains limited. Therefore, the aim of this article is to explore how representatives of the equine sector—and riding schools in particular—conceptualize and articulate their work with environmental sustainability in policy and practice.

## 2 The Swedish riding schools

In Sweden, organizations within the sports and outdoor recreation sectors constitute an active and resource rich part of civil society, with considerable potential to assume a leading role in advancing a more sustainable society. The Swedish National Sports Confederation (SNSC), founded just over a century ago, functions as an umbrella organization and currently comprises 72 national sports federations, which together include approximately 19,000 sports associations. The Swedish Equestrian Federation (SEF) is one of these federations, and approximately 430 riding schools are affiliated with SEF. The ownership structures of these riding schools vary considerably: buildings and land may be owned by different stakeholders, both private and public (15). The riding schools are governed by an elected board. However, the everyday activities are managed by a director who is either employed by the board or by a privately owned company. Like other sports clubs, riding schools receive public financial support for the organization of activities for children and young people.

For most horse riders in Sweden, riding schools constitute their first point of contact with horses and equestrian sports, often beginning in childhood. Riding schools primarily offer services such as riding lessons for both children and adults, along with opportunities for participants to care for, learn about, and spend time with horses. In addition, they generate demand for agricultural products including hay, oats, straw, and wood shavings (16, 17).

## 3 Framework

This study draws on two complementary frameworks. First, environmental sustainability is understood in relation to the broader policy terminology of sustainable development, as articulated in the Brundtland Report (18), Agenda 2030, and the Sustainable Development Goals (SDGs). These provide an important societal and policy context for understanding how environmental sustainability is defined and promoted within contemporary organizations, including the equine sector.

Second, the study is informed by ecolinguistics, particularly Stibbe's (19) concept of “stories we live by”. While the sustainable development framework helps identify the environmental themes and goals that are prioritized, ecolinguistics provides analytical tools for examining how sustainability is conceptualized, communicated, and given meaning through language. Together, these perspectives enable us to explore both the content of sustainability work and the narratives through which environmental responsibility is understood and enacted within Swedish riding schools.

We therefore analyze the empirical material at two interconnected levels. First, we examine the extent to which policy documents and interviewees relate to established sustainability concepts, particularly those associated with Agenda 2030 and the SDGs. Second, we examine how environmental sustainability is linguistically framed and what recurring narratives, or “stories we live by”, emerge in descriptions of environmental responsibility, learning, and action.

The concept of sustainable development is articulated as follows in the Brundtland Report (18):

…development that meets the needs of the present without compromising the ability of future generations to meet their own needs.

While the three dimensions of sustainable development—economic, social, and environmental—are interconnected and mutually influential, the focus of our study is on environmental sustainability. To provide an overview of how sustainability work is conceptualized at riding schools in Sweden, we relate our findings to the broader context of sustainable development, Agenda 2030, and the Sustainable Development Goals (hereafter SDGs). These frameworks are not scientific or theoretical constructs, nor are they intended to function as analytical tools. Rather, they operate as policy instruments. It is therefore of interest to examine whether, and in what ways, they inform or shape sustainability terminology in specific contexts—in this case, at Swedish riding schools. We suggest that the stronger the thematic and conceptual alignment between a riding school's environmental work and official sustainability documents and initiatives, the more the riding school can be understood as participating in, and committed to, the broader societal sustainability efforts and terminology. When a riding school employs concepts and themes associated with the sustainable development terminology, we interpret this as evidence of an active and engaged incorporation of the knowledge, priorities, and policy frameworks embedded in that terminology. Such engagement may, in turn, suggest that the riding school's environmental work is informed by an awareness of both the significance and the complexity of contemporary sustainability challenges. Conversely, when the concepts and themes used do not align with the sustainable development terminology, the riding school's environmental efforts are likely characterized by a lower degree of such awareness.

In this study, this approach is supported and further informed by research in ecolinguistics. The purpose of employing an ecolinguistic framework is not primarily to assess the environmental effectiveness of specific sustainability measures, but to examine how sustainability is conceptualized and linguistically framed. Following Stibbe (19), language is understood as shaping and reflecting the “stories we live by”, that is, recurring narratives that influence how people understand human-nature relationships and environmental responsibility.

Stibbe (19) demonstrates that language use related to environmental issues can both reflect and shape the awareness and engagement of individuals and organizations. Similarly, Cheng (20) argues that ecolinguistics can help reveal the relationships between linguistic practices and ecological awareness. Ecolinguistics, which has emerged from the broader ecological turn within the humanities and social sciences, examines the relationship between language and the environment, including their mutual influence. The field evaluates whether linguistic practices are eco-beneficial, eco-destructive, or eco-ambivalent, and seeks to promote ecological awareness and ultimately contribute positively to ecosystems. Stibbe (19) refers to “stories we live by”—the narratives constructed through language that shape how we perceive and engage with the world. Here we examine how environmental sustainability is linguistically framed in policy documents and interviews, and how these framings contribute to recurring “stories we live by” about environmental responsibility, learning, and sustainable development within Swedish riding schools.

## 4 Method

To enhance understanding of how ecological sustainability is conceptualized and articulated within the Swedish equine sector—and more specifically within riding schools—we employed qualitative research methods. These included a document analysis of policy texts addressing environmental sustainability and semi-structured interviews with representatives from ten different riding schools. All interviews and documents from the riding schools are anonymized. All quotations have been translated from Swedish to English; this applies to both the policy documents and the interview material.

The study was informed by investigator triangulation, whereby multiple researchers contributed to the collection and interpretation of the data (21). The researchers have diverse academic backgrounds, including philosophy, gender studies, history, sport science, and education, which provided a wide range of insights throughout the preparation, collection, and analysis of the material. Their levels of experience with the equine sector also varied. Three authors have long-standing experience within equestrian sports and riding school contexts, while two are newcomers to the field. This diversity contributed to a deeper understanding of what is specific to the equine sector in relation to other sporting activities.

### 4.1 Policy documents

The policy documents were retrieved from national level equine organizations as well as from riding schools. The national organizations included the Swedish Horse Industry Foundation, the Swedish Horse Industry Representative Council, Swedish Harness Racing, the Swedish Equestrian Federation, Lövsta Future Challenge, and the Federation of Swedish Farmers—Horse. The riding schools were selected based on their membership in the Swedish Equestrian Federation (SEF), as well as their size and geographic location. In 2022, SEF had 841 member clubs, 450 of which were riding schools. We compiled an Excel list of these schools and sorted them by location and used the Hästnäringen i siffror (22) (“The Equine Sector in Numbers”) database to categorize them according to the number of horses and members. Based on these criteria, the riding schools were grouped into large, medium sized, and small categories. We then contacted 30 riding schools and invited them to participate in interviews about their sustainability work. We received answers from nine big riding schools, whereas only one of the medium sized answered and participated. None of the small riding schools answered, even though they were reminded several times. The national documents were easily accessible through various organizational websites. Retrieving the local documents proved more challenging. Although many respondents at the local level expressed a willingness to share their documents, obtaining them in practice was considerably more difficult. After reviewing websites and sending several reminders, we were able to secure five riding school policies (Supplementary Appendix S1), which constitutes the material used for this research.

### 4.2 Interviews

In total, we conducted ten semi-structured interviews with board members and staff from Swedish riding schools. The interviews were carried out either via Zoom or in person at the stable, depending on the interviewee's preference. Onsite interviews were held in a quiet room without other participants to ensure confidentiality and anonymity. All interviews were audio recorded and transcribed (Supplementary Appendix S2).

The interviews were conducted between August and December 2022 by one or two members of the research team. When interviews were carried out by a single researcher, this person had extensive experience with qualitative interviewing as well as prior knowledge of the equine sector. When conducted by two researchers, we ensured that the pair was composed so that one had substantial methodological expertise and the other was familiar with the equine sector, or vice versa.

General questions about the riding school were posed, such as: “Can you tell me about the riding school and the work you are engaged in?” Questions related to environmental work included: “How do you perceive the environmental challenges within the equine sector?”, “Does the riding school have any environmental or sustainability policies?”, and “Does the organization allocate resources—financial, personnel, knowledge, or expertise—for environmental initiatives?” These were followed by future oriented questions such as: “What do you hope the equine sector will look like in 10 or 50 years?” and “What should a sustainable equine sector look like in the future?”

### 4.3 Analysis

The analysis was conducted as an abductive qualitative thematic analysis, moving iteratively between the empirical material, the research questions, and the theoretical concepts drawn from sustainable development and ecolinguistics (19). The purpose was both to identify how environmental sustainability was addressed in documents and interviews and to explore how sustainability was linguistically framed and understood within the riding school context.

### 4.4 The analysis was carried out in four stages

First, all policy documents and interview transcripts were read repeatedly by all members of the research team to gain familiarity with the material. During this phase, preliminary observations were made regarding sustainability-related concepts, recurrent themes, and patterns of meaning.

Second, the material was coded using a combination of deductive and inductive coding. Deductive codes were derived from the research questions and included references to sustainability terminology, environmental goals, environmental practices, learning, responsibility, and future visions. Inductive coding was subsequently used to identify recurring patterns emerging from the material itself, such as manure management, resource constraints, repair and reuse practices, role modelling, and organizational support.

Third, the coded material was analyzed in relation to the three research questions:

**RQ1**. To what extent and in what ways do the documents and interviewees relate to the SDGs or the sustainability terminology more broadly, and are specific environmental goals articulated?

For this question, we examined explicit and implicit references to sustainable development, Agenda 2030, the SDGs, environmental responsibility, and sustainability-related goals.

**RQ2**. What local practices related to sustainability terminology or the SDGs are described?

Here, we identified descriptions of concrete sustainability practices and examined how these practices were discussed and justified by respondents.

**RQ3**. How is sustainability as a learning area addressed in the policy documents and in the interviews?

In relation to this question, we analyzed how knowledge, learning, teaching, role modelling, and educational ambitions connected to environmental sustainability were constructed in both documents and interviews.

Fourth, the material was interpreted from an ecolinguistic perspective. Following Stibbe (19), particular attention was paid to how environmental responsibility, agency, learning, and relationships between humans and nature were linguistically framed. We explored whether the language used could be interpreted as predominantly eco-beneficial, eco-ambivalent, or eco-destructive, and we identified recurrent narratives, or “stories we live by”, concerning sustainability within the riding school sector. These narratives were not understood as fixed categories but as recurring ways of making sense of environmental sustainability across documents and interviews.

Finally, the codes and interpretations were discussed among the researchers and grouped into broader analytical themes. Through this process, a number of recurring sustainability narratives emerged, which form the basis for the discussion section.

The codes were used as analytical tools rather than fixed categories and were continuously refined throughout the abductive analysis. An overview of the coding scheme and the main analytical categories is provided in Supplementary Appendix S3.

We chose not to address how riding school students and staff travel to the riding schools even though such transport practices are frequently highlighted in discussions on how to make equestrian sports more environmentally sustainable. This delimitation is motivated partly by the fact that the topic of transportation is examined more thoroughly in Hedenborg et al. (23), and partly because riding schools have limited authority over broader transport infrastructure in society.

## 5 Results

### 5.1 SDGs and the sustainability terminology in national level policy documents

In this section, we examine how the policy documents and interviewees relate to the SDGs and to the sustainability terminology more broadly. One might expect that the higher the organizational level behind a policy document, the more closely it aligns with the established terminology on sustainable development. Such a pattern would not be surprising, given that national level documents are typically produced by individuals more extensively engaged in sustainability issues and likely possessing some degree of expertise in the area.

By contrast, the policy documents produced by local riding schools represent only a small part of their overall operations, and their primary competences and daily concerns lie outside the domains of sustainable development and environmental management. As will be shown below, our expectations were, to some extent, confirmed. The Swedish equine sector's national strategy, the National Horse Industry Strategy (24), refers explicitly and closely to Agenda 2030 and to the SDGs, stating:

… below is briefly described examples of how the horse sector contributes to Sweden's environmental goals and Agenda 2030—the global goals for sustainable development. The overall goal in the national environmental work is to hand over to the next generation a society where the major environmental problems are solved, without causing increased environmental and health problems outside Sweden's borders.

In the second part of the quotation, there is also a reference to the definition used by the Brundtland Commission. The National Horse Industry Strategy further specifies which SDGs are most relevant to environmental sustainability in Swedish riding schools (25):

SDG 7: Affordable, reliable, sustainable and modern energy
SDG 12: Responsible consumption and production
SDG 13: Climate action
SDG 15: Life on land (ecosystems and biodiversity)

In addition, the text identifies several other SDGs as relevant for the equine sector more broadly, including SDG 3 (Good health and wellbeing), SDG 4 (Quality education), SDG 8 (Decent work and economic growth), and SDG 11 (Sustainable cities and communities).

Policy documents at lower organizational levels do not relate to the Bruntland Report, the Agenda 2030 nor SDGs or the sustainability terminology in the same explicit manner. The SEF even employs an uncommon and unestablished terminology in one of its documents, stating that it seeks sustainable development on the “terms of nature” (26). This phrase is not part of the established sustainable development terminology, and its use indicate a weaker connection to the SDGs. However, another strategic document from SEF, the Sustainability Strategy (27), clearly connects to the SDGs. The following six SDGs are identified as particularly relevant for equestrian sports:

SDG 3: Good Health and Well-Being
SDG 5: Gender Equality
SDG 10: Reduced Inequalities
SDG 13: Climate Action
SDG 14: Life Below Water
SDG 15: Life on Land

For the purposes of this study, SDGs 13, 14, and 15 are of direct relevance. The Sustainability Strategy also articulates the ambitious goal that “everyone in equestrian sports shall work actively with sustainability in 2025” (27, p. 6). In the document, the SDGs are operationalized for the local context. For example:

SDG 13 (“Take urgent action to combat climate change and its impacts”) becomes: “We actively work to reduce our carbon footprint.”
SDG 14 (“Conserve and sustainably use the oceans, seas and marine resources”) becomes: “We actively work to reduce water usage and eutrophication.”
SDG 15 (“Protect, restore and promote sustainable use of terrestrial ecosystems…”) becomes: “We contribute to increased biodiversity.”

The phrase “on nature's terms” appears in this document as well, used as the headline for the section addressing SDG 14 and SDG 15.

Two of the national level documents aim explicitly at adapting environmental practices in riding schools. The first is the Environmental Check (28), produced by SEF and Lövsta Future Challenge. It is a checklist for organizing “green” competitions and consists of 20 “climate measures”, grouped into five themes: transportation, recycling, purchasing, food service, and manure and water management.

The other national level document is the Equine Sector's Quality Label (29), which is overseen by the Swedish Horse Industry Foundation and the Federation of Swedish Farmers—Horse. Although the Quality label encompasses more than environmental aspects, the environmental component is grounded in current legislation and stresses the importance of compliance. All certified businesses undergo regular inspections. In addition to its role in the Quality label, the Federation of Swedish Farmers has its own environmental policy document (30), which states:

Ultimately, the development towards a sustainable society must take place at the companies, not through laws and regulations.

This statement may appear somewhat surprising, particularly in the context of a certification scheme that is largely based on legal compliance.

In summary, the national-level documents display a strong alignment with the established sustainability terminology associated with the Brundtland Report, Agenda 2030, and the SDGs. References to global sustainability goals are explicit, and environmental objectives are translated into sector-specific ambitions relating to climate action, biodiversity, water quality, and resource use.

From an ecolinguistic perspective, these texts do more than communicate environmental priorities. They construct sustainability as a shared and active responsibility. Formulations such as “we actively work” and “we contribute” position organizations and their members as agents capable of influencing environmental outcomes. The repeated use of collective and action-oriented language suggests sustainability as both achievable and desirable. Following Stibbe (19), such language may be interpreted as containing eco-beneficial elements because it promotes engagement, responsibility, and care for ecological systems.

The documents also reveal a particular way of understanding environmental sustainability. Rather than emphasizing compliance alone, they frame sustainability as an ongoing collective commitment. As such, the national policy documents contribute to what might be described as a sustainability-oriented story to live by, in which riding schools and equestrian organizations are expected to act as active contributors to environmental change rather than passive recipients of regulations.

### 5.2 SDGs and the sustainability terminology in policy documents at the local level

In this section, we turn to the policy documents produced by local riding schools. To begin with, it is important to note that several of the riding schools we contacted did not appear to have any sustainability strategy in place. In this sense, they can be understood as being positioned far from the established sustainability terminology. Among the documents that were available, two appear to be based on a common template. Certain phrases recur verbatim, while other sections seem to originate from a shared source but have been modified in slightly different ways. Overall, the concepts employed in these local documents are situated further from the sustainability terminology and the SDGs than those found in policy documents produced at the national level.

Some documents nevertheless demonstrate a degree of familiarity with the broader sustainable development terminology, committing to strategies such as:

To appoint a sustainability resource to assist the riding school's manager's environmental efforts (Policy Document 1).

Riding schools are, as noted above, governed by a board, and this board holds overall responsibility to ensure that sustainability efforts permeate the everyday work of the organization. The quotation above illustrates how the board can support such efforts, but the operationalization of sustainability work is delegated to the riding school manager. The manager is further responsible for:

To clearly and positively communicate the work and goals for sustainable development with the staff, elected officials, volunteers, and all club members through the website, notices in the stable, and at the facility (Policy Document 1).

In this case, the policy document explicitly refers to the organization Keep Sweden Tidy (31), a national foundation that promotes recycling and combats litter through public awareness campaigns and environmental education. Policy Document 1 is also the only local level document that articulates a specific goal which relates to other sustainable development documents:

Our goal is to obtain the Swedish Equestrian Federation's environmental certification for sustainable events and competitions, and environmental certification through Keep Sweden Tidy (Policy Document 1).

The certifications mentioned here likely refer to the Equine Sector's Quality Label and the Environmental Check, both of which are tools designed to support environmentally sound practices—for instance, in the organization of “green” competitions. The reference to an environmental certification from Keep Sweden Tidy is, however, somewhat unclear, as the foundation states that no such certification exists (32).

Other documents exhibit a weaker connection to the sustainable development terminology but still signal some degree of alignment. For example, one strategy for achieving environmental goals is articulated as:

… organize competitions in a sustainable way (Policy Document 2).

There are also documents that show even less connection to the sustainability terminology, instead framing their environmental work in terms of internal organizational values rather than established sustainability concepts:

Our core values are based on three cornerstones: knowledge, openness and community, and these reflect the ambition of our environmental work (Policy Document 3).

How knowledge, openness, and community are intended to support environmental work is not articulated, although this may reflect an ambition to learn more about how such work can be developed. Another example of how sustainable development is articulated can be found in a document that emphasizes the need for environmental work to be grounded in legislation:

Environmental work must comply with environmental legislation, and it must form the basis of our environmental work (Policy Document 4).

This document further highlights the importance of continuous improvement:

Sustainability work regarding the environment is characterized by continuous improvement, for example through an effort to reduce energy use and to reduce use of resources (Policy Document 5).

In summary, the local policy documents display a weaker connection to the established sustainability terminology than the national-level documents. References to Agenda 2030, the SDGs, or broader sustainability frameworks are rare, and environmental ambitions are more often framed through organizational values, everyday responsibilities, compliance with legislation, or practical improvements. Sustainability is therefore articulated in a more local and practice-oriented manner than in the national policy documents.

From an ecolinguistic perspective, this difference is significant. While the national documents construct sustainability through explicit reference to global goals and established sustainability concepts, the local documents tend to frame environmental responsibility as something that emerges through everyday management, learning, and organizational routines. Sustainability is less often presented as participation in a global transition and more often as a matter of providing conditions for responsible local action.

The language of the local documents is therefore difficult to categorize as clearly eco-beneficial or eco-destructive. Rather, it is characterized by varying degrees of eco-ambivalence. Environmental responsibility is generally viewed positively, yet the ecological rationale is often implicit rather than explicitly articulated. At the same time, several formulations emphasize engagement, learning, and practical action, suggesting emerging eco-beneficial elements. Taken together, these documents point towards a recurring story in which sustainability is understood primarily as something that is learned and enacted through everyday organizational practice rather than through formal adherence to sustainability frameworks.

### 5.3 Relation to the SDGs or to the sustainability terminology in interviews at the local level

The analysis of the interviews shows that the relationship to the SDGs and the broader sustainability terminology is weaker in the interview material than in the policy documents. In the spoken material, established sustainability concepts rarely appear, which can partly be explained by the fact that such terminology is more typical of written documents than of everyday speech. At the same time, interviewees express an awareness of differences in environmental commitment between non-profit riding schools and more professionalized sectors, suggesting a perception that riding schools are lagging in the broader societal transition toward sustainability. The interviewees highlighted a perceived discrepancy between environmental awareness in professionalized sectors—such as the private corporate sphere—and in riding schools, which typically operate as non-profit associations. One interviewee, who also worked at a hotel with environmental certification, drew the following comparison:

Environmental awareness within the hotel and restaurant industry is strong. I'm currently working at, or have worked at, a hotel certified with the Nordic Swan Ecolabel. …//… What we focus on at the riding school, or what I primarily think about there, is what kind of materials we use for cleaning. Then there's the feed—how people we buy the feed from are working with sustainability … and the same goes for wood shavings, which are currently a bit of a scarce resource (Riding School 5).

During the interviews, we asked whether the riding school had an environmental policy and whether it was used in practice. The existence and perceived usefulness of policy documents varied considerably. In several cases, the documents did not appear to be integrated into everyday work. One interviewee described how the policy had merely served as a “shelf warmer” until a sustainability group was established:

And I was so happy to feel that I didn't have to be the only one thinking about the environment and sustainability… In any case, we are either going to update that policy or create a completely new one (Riding School 2).

Another noted:

I'll be honest and say that I haven't really looked at it myself, and it's completely non-existent within the board, I'd say (Riding School 3).

Some riding schools reported not having any environmental policy at all, though they still expressed awareness of the need to strengthen their environmental work:

But with the environment, we haven't come that far. We've sort of been stuck in this old loop of not thinking much about it. But it's an area where we're really weak. And we really do need to improve the environment (Riding School 6).

One interviewee pointed out that policy documents are not in themselves a guarantee of good practice, yet this same interviewee explicitly referred to Agenda 2030:

This year, we will implement the Agenda 2030 goals in our practices… We want to make use of those beautiful labels that Agenda 2030 has. Then we can work with them in a concrete way. The Swedish Equestrian Federation has made a little booklet with six sub-goals, I think. But we'll review that. I believe we have slightly higher ambitions than the Swedish Equestrian Federation (Riding School 10).

We were also interested in whether the riding schools had articulated specific goals for their environmental work. The responses reveal a wide variation. Recurring themes include manure management, water usage, electricity, littering, and waste sorting. Many interviewees expressed strong ambitions and demonstrated awareness of complex issues such as eutrophication. Manure and nutrient leakage were recurring concerns:

We have high ambitions, and we want to contribute in a good way. So now we're discussing water collection, we're discussing solar energy, and we're talking about how we can manage horse manure and so on. It's constantly on the agenda. We realized that we had a phosphorus trap… For our paddock, there's a phosphorus risk in relation to a lake (Riding School 1).

Not all riding schools were satisfied with their environmental efforts, however:

I would say there's a complete absence of this issue at the riding school. They just keep going like they always have… The first goal really needs to be to get waste sorting in place. That feels like a small, manageable step (Riding School 3).

Waste handling—particularly plastic from silage and bedding—was a recurring challenge. Several riding schools also discussed lighting upgrades and the potential for installing solar panels. Sustainable purchasing was not a dominant theme, but some schools emphasized long-lasting equipment. One interviewee expressed the desire for more dedicated sustainability roles.

I wish I could allocate a few more hours for someone on the staff to work on environmental issues… We don't have anyone whose job description includes environmental work, but I would really like to see that happen (Riding School 10).

Local practices ranged from centrally supported initiatives—such as waste sorting and the Environmental Check—to creative bottom-up approaches like repairing blankets and transforming old materials into new items and consumption emerged as a significant theme:

There's a sort of material obsession… Maybe the ideal isn't having 10–20 saddle pads… Instead of constantly buying new things (Riding School 8).

Some schools organized clothing swap days, second-hand shops, or annual flea markets. Avoiding single-use items in the cafeteria was another small but meaningful step.

In summary, the interviews reveal a relatively weak connection to the formal sustainability terminology associated with Agenda 2030 and the SDGs. Explicit references to sustainability frameworks are rare, and many respondents describe environmental work without using the terminology commonly found in policy documents. At the same time, the interview material demonstrates considerable engagement with environmental issues and a strong willingness to improve current practices.

From an ecolinguistic perspective, the language used by the interviewees is characterized less by references to abstract sustainability goals and more by narratives of responsibility, aspiration, and constraint. Environmental sustainability is frequently discussed through practical concerns such as manure management, waste sorting, energy use, and consumption, rather than through broader environmental concepts. The interviewees position themselves as actors who want to contribute positively but who often lack sufficient resources, knowledge, or organizational support to achieve their ambitions.

This recurring way of talking about sustainability is neither eco-destructive nor strongly aligned with established sustainability frameworks. Rather, it reflects a predominantly eco-ambivalent narrative containing several eco-beneficial elements. Interviewees consistently express environmental concern and describe concrete actions, yet they simultaneously portray sustainability work as fragmented and incomplete. Taken together, these accounts point towards a recurring story in which riding schools view themselves as willing participants in environmental change but as actors who cannot achieve this change alone. The dominant narrative is therefore not one of resistance to sustainability, but one of commitment constrained by limited capacities and support structures.

### 5.4 Sustainability as a learning area in policy

In this results section, we examine whether, and in what ways, riding schools include education for sustainable development in their pedagogical work. We analyze when—and when not—sustainability is articulated as an area for learning at both the national and local levels.

At the national level, the Horse Industry Representative Council explicitly emphasizes learning:

Horse owners and horse entrepreneurs are offered information on climate and energy issues. Their motivations, needs, and interests should guide this work. To accelerate the transition, action is required by individual horse keepers and horse entrepreneurs. In these processes, knowledge and decision-making support are needed. It is a shared task, among other things, for authorities and organizations within the horse sector to collaboratively develop relevant decision-making and knowledge support (30).

The National Horse Industry Strategy communicates a similar message, highlighting the need for sustained knowledge development to address environmental challenges:

Society, the horse sector, and horse businesses constantly need new knowledge to develop and meet external expectations and societal challenges. The horse sector needs to provide and disseminate new knowledge, and still valid old knowledge based on scientific methods or experience. Competence provided to the horse sector and horse businesses through education and advice must meet the needs of horse businesses and adapt as these needs change based on external factors and the development of operations (24).

In contrast, the other national policy document from SEF do not explicitly address learning.

As already mentioned, at the local level, two riding schools appear to use a shared template for their environmental policy documents, with modifications likely based on local conditions. This pattern also applies to how learning and sustainability are discussed. Three policy documents, using identical wording, state an ambition to:

… provide employees and students with education and information and create conditions so that everyone can engage in active and visible environmental work (Policy Document 1, 4, & 5).

Policy Document 3 similarly emphasizes the importance of awareness, knowledge, and education for reducing environmental impact.

Taken together, these findings suggest that learning is given more attention in local policy documents than at the national level. Interviewees also express a desire for SEF to be more supportive in relation to how learning and sustainable development can be related to the local level challenges:

The Swedish Equestrian Federation also works according to the global sustainability goals. And if you attend various lectures or forums, that's the kind of information we are fed with from the central level. Which, of course, we take in and want to… well, it should trickle down into our system, into our club and such. And of course, it then becomes much more operational. It's not always easy to relate to, but I think that work must continue (Riding School 1).

The need for both inspiration and knowledge among staff to enable teaching about sustainability was highlighted. At one riding school, staff had participated in an educational and inspirational study visit to other riding schools more advanced in their sustainability work. This visit provided knowledge about issues such as nutrient leakage and local water management. One interviewee stressed the importance of integrating environmental education in horse-related training programes:

I really wish that environmental education was a more natural part of all equestrian training programs. It shouldn't be something where people go “ugh, now we must do the environment too”, but rather… it should feel natural. And then, when people are eventually employed, it should be integrated into your way of working to think in a certain way (Riding School 10).

One interviewee pointed out the vulnerability associated with relying on one or two particularly engaged individuals: if these individuals leave, sustainability work risks coming to a halt.

The interviews reveal several arenas for environmental learning at riding schools. Interviewees simultaneously emphasized their own role—as well as that of other staff, riding instructors, and even parents—as role models for students. This includes demonstrating everyday sustainable practices such as waste sorting:

And I understand that if we don't sort our waste, it may not seem like a big deal, but it sends a very strong signal, I think, that we do it. Especially since we have so many children moving around the premises. I think it's important to show that, yeah, this is something we care about… (Riding School 3)

Parents may also contribute by reinforcing sustainable behaviours when accompanying younger students. Furthermore, many riding schools have youth sections where young people can propose and implement ideas, and interviewees stressed the importance of collaboration with these groups. Conversations about energy and water conservation—such as turning off lights, conserving heat, and preventing water wastage—were frequently mentioned:

We provide a lot of information. We have a lot of signs posted, especially about lighting—reminders to turn off lights, to clean the wash stalls, not to overuse water when rinsing off horses, and to check the automatic waterers so they're not constantly leaking (Riding School 5).

Motivations for these initiatives vary, ranging from environmental concern to economic considerations. Nonetheless, riding schools consistently present sustainability as an area of shared responsibility, communicated through both verbal guidance and signage in the stable environment.

In summary, sustainability is addressed as a learning area in both policy documents and interviews, although the forms and ambitions of such learning vary considerably. National and local policy documents emphasize the importance of knowledge development and environmental awareness, while the interviews reveal that sustainability-related learning primarily takes place through everyday activities, social interaction, and role modelling rather than through structured educational programes.

From an ecolinguistic perspective, the interview material constructs sustainability as a shared responsibility that is learned through participation in the everyday life of the riding school. Environmental practices such as waste sorting, reducing water use, conserving energy, and reusing materials are communicated not only as practical measures but also as examples of the values that riding schools wish to promote. The repeated emphasis on role models, youth engagement, and collective responsibility suggests that sustainability is understood as something that is acquired through observation and participation as much as through formal instruction. At the same time, the material reveals a lack of common pedagogical structures and clearly articulated educational goals related to sustainable development. Learning therefore appears dependent on the commitment and competence of individual staff members rather than being systematically embedded within riding school activities. This creates a degree of eco-ambivalence: sustainability is widely regarded as important, yet the mechanisms through which environmental knowledge should be taught and sustained over time remain relatively underdeveloped.

Taken together, the documents and interviews point towards an emerging story in which riding schools function as informal learning environments for sustainability. In this story, environmental responsibility is transmitted through everyday practices, social relationships, and role modelling, suggesting that sustainability is learned primarily through participation in the culture of the riding school rather than through formal educational frameworks.

## 6 Discussion

The aim of this study was to explore how representatives of the equine sector, and riding schools in particular, conceptualize and articulate environmental sustainability in policy documents and everyday practice. Drawing on Stibbe's (19) concept of “stories we live by”, we interpret our findings as revealing a set of recurring sustainability narratives that shape how environmental responsibility is understood and enacted within Swedish riding schools. These stories are not always explicitly expressed through the language of Agenda 2030 or the SDGs. Rather, they emerge through policy formulations, interview accounts, and descriptions of everyday practices. Together, they illuminate how sustainability is given meaning within the riding school context.

### 6.1 Story 1: the responsible local actor

A first and recurring story portrays the riding school as a responsible local actor. This story appears in both policy documents and interviews and is expressed through references to manure management, nutrient leakage, biodiversity, water conservation, and energy consumption.

Unlike the national policy documents, local actors rarely refer explicitly to the SDGs. Nevertheless, many interviewees describe concrete environmental responsibilities associated with caring for horses, land, and surrounding ecosystems. Environmental sustainability is therefore framed as a practical and moral responsibility connected to everyday horse keeping rather than as compliance with global sustainability frameworks.

From an ecolinguistic perspective, this narrative can be interpreted as predominantly eco-beneficial because it positions environmental care as desirable, necessary, and compatible with the core mission of riding schools. Rather than constructing nature as a resource to be exploited, the language frequently emphasizes care, responsibility, and long-term management. The story that emerges is one of agency, where riding schools understand themselves as custodians of both horses and local environments.

### 6.2 Story 2: we try, but we lack resources

A second narrative centers on ambition combined with constraint. Across the interviews, environmental sustainability was consistently described as important and desirable. However, respondents simultaneously emphasized limited resources, insufficient knowledge, fragmented ownership structures, and a lack of organizational support.

This story differs from narratives of environmental denial or resistance. Interviewees rarely questioned the importance of sustainability. Instead, they frequently described barriers to acting on their ambitions. Sustainability was presented as something that should be pursued, but that requires additional expertise, financial resources, time, and institutional support.

The narrative reveals a perceived gap between expectations and capacities. Many interviewees expressed a need for stronger guidance from sector organizations and more opportunities for learning and exchange. In this sense, sustainability work appears characterized by what might be termed institutional loneliness. Environmental responsibility is acknowledged, but many riding schools experience themselves as relatively isolated actors attempting to address complex challenges with limited support structures.

From an ecolinguistic perspective, this story can best be understood as eco-ambivalent. The underlying values are clearly supportive of environmental sustainability, yet the discourse simultaneously constructs sustainability as difficult to realize because responsibility is dispersed and capacities are constrained.

### 6.3 Story 3: learning through everyday practice

A third story concerns sustainability as a learning process. Although environmental education is only weakly institutionalized in most riding schools, sustainability is nevertheless communicated and reproduced through everyday practice.

Interviewees repeatedly emphasized the importance of role modelling. Riding instructors, staff members, board representatives, and parents were all described as influencing how young people learn to relate to environmental issues. Waste sorting, energy conservation, water management, and reuse practices were not only environmental measures but also educational practices through which norms and values were communicated.

This finding is particularly interesting given the educational role historically associated with Swedish riding schools. Environmental learning rarely appeared in the form of structured curricula or formal educational objectives. Instead, it was embedded in routine activities and social interactions. Sustainability therefore emerged as something learned through participation rather than instruction.

Viewed through Stibbe's framework, this narrative reveals an emerging sustainability story that is transmitted through practice and social relations. The story suggests that environmental responsibility is not merely something people are told about but something they learn by observing and participating in everyday activities.

### 6.4 Story 4: sustainability without sustainability language

While national policy documents display a strong alignment with Agenda 2030 and established sustainability terminology, the interviews reveal much weaker connections to the established terminology.

However, the absence of SDG language should not be interpreted as an absence of sustainability work. On the contrary, interviewees described numerous initiatives aimed at reducing environmental impacts, including waste management, reuse, energy efficiency, and water conservation. Sustainability was therefore often enacted without being explicitly labelled as sustainability.

This finding helps explain the apparent discrepancy between the document analysis and the interview material. National organizations appear more integrated into international and national sustainability discourses, whereas local actors largely draw upon practical knowledge and everyday experience. The result is a situation in which sustainability practices are often more developed than sustainability language.

According to Stibbe (19), “stories we live by” are created and reproduced through language. Our findings suggest that the sustainability stories present within riding schools are only partially articulated. The language has not yet fully caught up with practice. Consequently, some of the environmental work undertaken by riding schools remains difficult to communicate, coordinate, and scale beyond local contexts.

### 6.5 Story 5: small actions can make a difference

A final narrative concerns the significance of small-scale actions. Several interviewees described initiatives such as repairing horse blankets, organizing clothing swaps, establishing second-hand shops, reducing waste, and encouraging moderation in consumption.

From a systems perspective, such measures may contribute less to reducing environmental impacts than larger structural interventions. Nevertheless, these practices are important because they are visible, achievable, and meaningful within the local context. They also create opportunities for participation and engagement.

This narrative presents sustainability as something that can be integrated into everyday life rather than as a distant policy agenda. The emphasis is placed on practical action, creativity, and collective responsibility. Through these activities, sustainability becomes associated with opportunity, competence, and shared values rather than with sacrifice alone.

From an ecolinguistic perspective, this story contains strong eco-beneficial elements because it normalizes environmentally responsible behaviour and embeds it within the everyday culture of the riding school.

Taken together, these stories suggest that Swedish riding schools are neither environmental leaders nor environmental laggards. Rather, they are organizations in transition, attempting to integrate sustainability into their everyday activities while lacking a fully developed sustainability discourse.

The dominant story that emerges is therefore not one of environmental neglect but one of committed local actors seeking to act responsibly within the constraints of their organizational conditions. Sustainability is primarily expressed through agency, learning, practical engagement, and incremental change. The ecolinguistic analysis further suggests that eco-beneficial stories are already present within the riding school sector, although they remain fragmented and only partially articulated.

Strengthening the connection between practice and discourse may therefore be an important next step. Greater access to organizational support, pedagogical resources, and sustainability-related language could enable riding schools not only to undertake environmental action but also to communicate, teach, and expand the sustainability narratives that are already emerging in practice.

### 6.6 Limitations and avenues for future research

This study is limited by the availability and uneven quality of local policy documents and by a restricted sample of riding schools. Future research could profitably examine how different regional contexts and ownership models influence the sustainability trajectories of riding schools and other equine establishments, thereby shedding light on the governance arrangements that enable or constrain environmental progress. Longitudinal studies of education for sustainable development interventions—such as role-model-based modules and their impact everyday practices—would further clarify how pedagogical efforts translate into behavioural change over time. In addition, applying fine-grained ecolinguistic discourse analysis would make it possible to trace how language and practice co-evolve when new forms of organizational support or sustainability tools are introduced. Finally, exploring lifecycle and systems perspectives, including feed production, bedding materials, plastic use, and transport logistics, could help identify sector-specific leverage points with potential to advance environmental sustainability within the equine sector.

Building on this, further research is needed to examine the equine sector's potential to act as an active agent in sustainable development—not only regarding the environmental dimension, but also the economic and social dimensions. With more developed and coordinated efforts in these areas, riding schools could help generate sustainability oriented “stories to live by” (in ecolinguistics terms), thereby strengthening both their contribution to sustainable development and their capacity as learning environments for education for sustainable development.

### 6.7 Concluding remarks

The equine sector—riding schools in particular—appears ready to act and is already engaged in practice-based sustainability. What is needed now is a shift from islands of creativity to systems of learning and terminology: connecting everyday practices to shared language, pedagogical structures, and enabling governance. With targeted support, the sector can lead in building eco-beneficial “stories we live by”, aligning what is already done with how it is named, taught, coordinated, and scaled.

Overall, riding schools require support to further develop their sustainability work and to become a more forward-looking actor in society's broader sustainability transition. How such support should be designed—and who should provide it—remains to be determined. To ensure both that riding schools can become more active sustainability actors, and that their environmental work (for example in relation to manure management) is not so inadequate that their very social license to operate is jeopardized, we argue that riding schools must engage more closely with municipal sustainability initiatives and with the broader sustainability terminology. This, however, presupposes that riding schools are invited into and recognized within these arenas.

## Data Availability

The data presented in this study are available on request from the corresponding author. The documents used can be found using our references, and transcriptions from the interviews are saved at the Malmö University server and not published OA because of ethical reasons related to protecting the anonymity of our interviewees.
