# Supplementary material for: Sustainability stories in Swedish riding schools: an ecolinguistic analysis of policy documents and interviews
Source: Front Sports Act Living. 2026 Oct 14;8:1821456. doi: 10.3389/fspor.2026.1821456 (PMC13713634; doi:10.3389/fspor.2026.1821456)
Supplement: Supplementary file 1 [file Datasheet1.docx]

**Appendix 1**

Environmental policy documents from national organizations and local riding schools

| **Document** | **Organization** | **Year published** |
| --- | --- | --- |
| The National Horse Industry Strategy | The Swedish Horse Industry | 2018 |
| The Horse Industry's Climate Policy | The Horse Industry Representative Council | 2015 |
| Environmental Policy | Swedish Harness Racing | 2023 |
| Environmental Check | SEF and Lövsta Future Challenge | 2021 |
| Sustainability Strategy | Swedish Equestrian Federation | 2021 |
| On nature's terms | Swedish Equestrian Federation | 2024 |
| The Equine Sector’s Quality Label | The Swedish Horse Industry and the Federation of Swedish Farmers – Horse | Not specified |
| Policy document 1 | Riding School | Not specified |
| Policy document 2 | Riding School | Not specified |
| Policy document 3 | Riding School | 2018 |
| Policy document 4 | Riding School | 2022 |
| Policy Document 5 | Riding School | 2020 |
