# Supplementary material for: Sustainability stories in Swedish riding schools: an ecolinguistic analysis of policy documents and interviews
Source: Front Sports Act Living. 2026 Oct 14;8:1821456. doi: 10.3389/fspor.2026.1821456 (PMC13713634; doi:10.3389/fspor.2026.1821456)
Supplement: Supplementary file 2 [file Datasheet2.docx]

**Appendix 2.**

Interviewees, organization, year interview and interview time

| **Interviewee** | **Organisation** | **Year** | **Time h:min** |
| --- | --- | --- | --- |
| Board member | Riding school 1 | 2022 | 01:10 |
| Riding school manager | Riding school 2 | 2022 | 00:42 |
| Board member | Riding school 3 | 2022 | 00:19 |
| Riding school manager | Riding school 4 | 2022 | 00:33 |
| Riding school manager | Riding school 5 | 2022 | 00:43 |
| Riding school manager | Riding school 6 | 2022 | 00:32 |
| Riding school manager and Board member | Riding school 7 | 2022 | 00:51 |
| Riding school manager | Riding school 8 | 2023 | 00:44 |
| Riding school manager | Riding school 9 | 2023 | 00:34 |
| Riding school manager and Board member | Riding school 10 | 2023 | 01:17 |
