# Supplementary material for: Sustainability stories in Swedish riding schools: an ecolinguistic analysis of policy documents and interviews
Source: Front Sports Act Living. 2026 Oct 14;8:1821456. doi: 10.3389/fspor.2026.1821456 (PMC13713634; doi:10.3389/fspor.2026.1821456)
Supplement: Supplementary file 3 [file Datasheet3.docx]

**Appendix 3. Overview of the Coding Scheme**

| **Code** | **Description** | **Example** |
| --- | --- | --- |
| Sustainability discourse | References to sustainable development, Agenda 2030, SDGs, environmental goals, or sustainability terminology | “Agenda 2030”, “sustainable development”, “climate action” |
| Environmental responsibility | Expressions of responsibility, care, stewardship, or environmental commitment | “We want to contribute in a good way” |
| Environmental practices | Descriptions of concrete sustainability measures | Waste sorting, manure management, solar panels, water conservation |
| Resource constraints | References to lack of resources, knowledge, authority, or support | “We would like to do more”, “we need more knowledge” |
| Learning and education | Sustainability framed as teaching, learning, role modelling, or knowledge development | Staff acting as role models, youth sections, educational activities |
| Future orientations | Visions and aspirations regarding the future of the equine sector | “What should a sustainable equine sector look like?” |
| Collective agency | Language emphasizing shared responsibility and joint action | “We actively work…”, “everyone should engage” |
| Eco-beneficial discourse | Language that promotes ecological responsibility and positive environmental action | “We contribute to increased biodiversity” |
| Eco-ambivalent discourse | Language expressing concern for sustainability but with unclear goals, limited commitment, or practical tensions | General statements about doing one's best without specific environmental aims |
| Stories we live by | Recurring narratives concerning sustainability, responsibility, learning, and environmental action | The responsible steward; learning through everyday practice |

Following the coding process, related codes were grouped into broader interpretative narratives (’stories we live by’) inspired by Stibbe (2021). For example, the codes *environmental responsibility*, *collective agency*, and *environmental practices* contributed to the narrative *The Responsible Local Care taker*, while *resource constraints* and *future orientations* contributed to the narrative *We Try, but Lack Resources*.
